# Beyond price: individuals' accounts of deciding to pay for private healthcare treatment in the UK

**DOI:** 10.1186/1472-6963-12-53

**Published:** 2012-03-07

**Authors:** Catherine Exley, Nikki Rousseau, Cam Donaldson, Jimmy G Steele

**Affiliations:** 1 Institute of Health and Society and Centre for Oral Health Research, Newcastle University, Baddiley-Clark Building, Richardson Road, Newcastle NE2 4AX, UK; 2 Institute of Health and Society Newcastle University, Baddiley-Clark Building, Richardson Road, Newcastle NE2 4AX, UK; 3 Yunus Centre for Social Business and Health, Level 3 Buchanan House, Glasgow Caledonian University, Cowcaddens Road, Glasgow G4 0BA, UK; 4 Institute of Health and Society, Centre for Oral Health Research, School of Dental Sciences, Newcastle University, Framlington Place, Newcastle NE2 4BW, UK

## Abstract

**Background:**

Delivering appropriate and affordable healthcare is a concern across the globe. As countries grapple with the issue of delivering healthcare with finite resources and populations continue to age, more health-related care services or treatments may become an optional 'extra' to be purchased privately. It is timely to consider how, and to what extent, the individual can act as both a 'patient' and a 'consumer'. In the UK the majority of healthcare treatments are free at the point of delivery. However, increasingly some healthcare treatments are being made available via the private healthcare market. Drawing from insights from healthcare policy and social sciences, this paper uses the exemplar of private dental implant treatment provision in the UK to examine what factors people considered when deciding whether or not to pay for a costly healthcare treatment for a non-fatal condition.

**Methods:**

Qualitative interviews with people (n = 27) who considered paying for dental implants treatments in the UK. Data collection and analysis processes followed the principles of the constant comparative methods, and thematic analysis was facilitated through the use of NVivo qualitative data software.

**Results:**

Decisions to pay for private healthcare treatments are not simply determined by price. Decisions are mediated by: the perceived 'status' of the healthcare treatment as either functional or aesthetic; how the individual determines and values their 'need' for the treatment; and, the impact the expenditure may have on themselves and others. Choosing a private healthcare provider is sometimes determined simply by personal rapport or extant clinical relationship, or based on the recommendation of others.

**Conclusions:**

As private healthcare markets expand to provide more 'non-essential' services, patients need to develop new skills and to be supported in their new role as consumers.

## Background

Delivering appropriate and affordable healthcare is a concern for policy makers everywhere. Decisions must be made about allocating - and balancing the supply and demand of - finite healthcare resources [1]. This often means rationing the availability of services as a result of limited supply and/or funding. One mechanism of addressing finite resources is to introduce some element of co-payment, where patients pay a contribution towards the costs of their treatment [2]. Additionally, publicly financed and private healthcare can coexist where individuals choose to pay for some treatments themselves, or through private health insurance. As policy makers and funders address the challenges of providing care for ageing populations with chronic conditions, in a context of finite resources and seemingly constantly evolving new technologies, it may be that more treatments become defined as 'additional' rather than 'essential', with individuals perhaps having to bear the costs themselves.

At present we know little about what influences people's decisions to purchase healthcare treatment privately, or of their experiences and perceptions of having to pay for some aspect of their own healthcare [3]. Purchasing a private healthcare treatment requires the individual to become adept at being both 'patient' and 'consumer', able to understand and appraise the benefits of a single treatment, judge the 'quality' of the provider, and decide if the price is worth paying. Individuals are familiar with acting as a 'consumer' when choosing and purchasing household goods or services, but may be less skilled when choosing different healthcare treatments or providers. In addition, deciding to pay for healthcare is likely to be particularly challenging in a setting where healthcare is most frequently provided through a universal healthcare system. In the UK most healthcare services are provided through the National Health Service (NHS), however, primary care dental service differs in that most adults make a financial, albeit capped, contribution towards the costs of their treatments. This well established mixed market provides an ideal setting to explore how those, familiar with paying something, decide whether or not to purchase expensive private treatments and allows us to examine how these personal healthcare decisions are made.

### Paying for healthcare: primary care dental services a UK example

As with other NHS services, primary care dentistry was initially free to all at the point of delivery. However, by 1951 a co-payment system for dental services was introduced to manage unprecedented demand; this remains the basis of the current subsidised system [4]. Although some receive free NHS dental care, most adults make a personal financial contribution towards their primary dental care treatment costs. However, in contrast to primary care medicine, in dentistry the same practitioner (or practice) will often provide both NHS and private treatments [5,6]. This is not unusual, in other countries with universal healthcare systems clinicians can choose to work a proportion of their time in private practice, and patients (or insurance companies) pay them directly for the care they provide [7]. However, paying for (or contributing towards) the costs of healthcare can affect people's perceptions of what they want and regard as being good value. A recent review of NHS dentistry in England found that when people make a significant contribution towards their healthcare, they "often expect something tangible and physical" for their money; they are satisfied with paying provided costs "are a good fit with expectations or previous estimates, and particularly if there is no sense of being 'ripped off'; or 'encouraged' to have unnecessary treatments" (p21) [4].

Dental Implant Treatments (DITs) are one example of a treatment that generally can *only *be purchased from a private provider; private dental insurance rarely covers the cost of DITs in the UK. For a single missing tooth, DIT involves implanting a titanium post, to which a prosthetic tooth is attached. For more extensive tooth loss a number of implants are placed to support either a removable prosthesis (Implant Supported Overdentures - ISODs) or a fixed prosthesis (implant supported bridgework), the latter generally requiring more implants at a resultant higher cost. Alternative treatments for partial and complete tooth loss are available through routine NHS primary care services (conventional bridgework or dentures), but DITs offer functional advantage over dentures, in that they are anchored into the jaw limiting unwanted movement, and unlike bridgework, need not involve damaging any remaining natural teeth. ISODs have been proposed as a *minimum *standard of care, for, those with no teeth [8,9]. However, even this less expensive treatment is only available through the NHS to a very small number of people with specific clinical need and delivered through secondary care. The overwhelming majority of people who want to have DITs have to pay themselves. The costs are high and vary significantly within patient groups and between providers. For context, the maximum contribution patients make for any NHS course of treatment in England is about £200, as a guide ISODs cost around £2,000 to £3,000 increasing to up to £10,000 for a fixed restoration, and sometimes more. As a result, many people are compelled to 'choose' a removable denture, as DITs are something they either cannot or will not afford.

The costs of DITs must be borne by the purchaser, meaning that they may appear to have more similarities to private cosmetic dental procedures, than to NHS treatment options like dentures. Indeed, DITs epitomise some of the current tensions and dilemmas within primary care dentistry in the UK, seemingly occupying a space on a continuum between 'functional necessity' and 'aesthetic need'; the exact definition subject to individual interpretation. For some people DITs may be the only treatment option they are prepared to countenance, but others regard them as an 'ideal' or even an unjustifiable 'luxury' when there is a long established and widely used technology (dentures) available through the NHS. DITs provide a lens through which to examine some of the complex decision making processes people engage in when considering whether to pay for healthcare treatments.

Drawing on healthcare policy, health economics and medical sociology this paper uses the exemplar of private dental implant provision in the UK to examine, in detail, what factors people consider when deciding whether or not to pay for a costly healthcare treatment for a non-fatal condition. In so doing, this paper seeks to add to our, previously limited, understanding of patients' engagement with the private healthcare market in the UK.

## Methods

This paper draws on qualitative data collected from semi-structured interviews with people who had considered paying for DITs in primary care. The data are part of a larger Medical Research Council funded project which received NHS research ethics committee and research and development approval prior to the commencement of data collection (a full discussion of the whole study is given in our protocol paper [10]). As little is known about how people make decisions to pay for private health care treatment an inductive approach was adopted, whereby data collection and analysis occurred concurrently. The study was conducted in the North East of England during 2007 and 2008. Purposive sampling was used and participants were recruited via primary care dentistry. Recruiting clinicians sent out information about the study to potential participants. Names and addresses of contacted patients were not released to the study team and no reminders were sent. Those potentially interested in participation returned a 'consent to contact' form to the team. NR followed these contacts up by telephone, and, if appropriate, made an appointment to conduct the interview (two people decided not to participate at this stage). Most people chose to be interviewed in their home, two were interviewed at the university and two at their place of work. Before the interview participants were able to ask any further questions and completed a consent form.

Focused interviews were used to examine critically and in detail people's views and experiences of deciding whether or not to pay for health care. Focused interviews are a particularly useful tool to employ in an area where relatively little is known, such as how people make decisions to pay for healthcare. They are flexible enough to allow interviewer and interviewee to explore issues that are pertinent to the individual person, but which may have not been anticipated in advance, thus enabling a fuller understanding of the processes at work to emerge. Interviews explored: individual's dental history; discussions with the dentist about DITs; what factors the person considered when deciding whether or not to proceed with treatment; and, if they did, their experience of the DITs.

All interviews were digitally-recorded, transcribed verbatim and anonymised. Thematic analysis based on the comparative method was carried out [11,12]. Data collection and analysis occurred concurrently; emergent themes and issues from earlier interviews informed the structure of subsequent ones. Data collection ceased when no new themes were being identified ('saturation'). CE and NR initially coded the data, and the wider research team participated in data sessions to discuss emergent codes. As data collection and analysis progressed, a coding frame was devised, tested and adjusted, and once refined applied to the transcripts using NVivo 7.

### Participants

39 people (16 men; 23 women; ages 23-84) were interviewed; the majority (n = 22) were over 60 years old (extensive tooth loss is associated with age); 12 were referred to secondary care for DITs. This paper takes as its focus the accounts of the remaining 27 respondents who *considered paying *for DITs in primary care: 10 paid for DITs privately, 14 did not and 3 when interviewed remained undecided. Participants came from very varied social backgrounds; current and (for those retired) previous occupations of those interviewed included a joiner, a clerical worker, a school teacher, and a self employed business man. In the paper, respondents quotes are identified in the following way: sex (M/F), date of interview, age and whether or not they paid for DITs (paid/declined) (egM030309;56;paid).

## Results and discussion

The analysis of these qualitative data suggest that the decision to pay for a private healthcare treatment is complex and mediated not only by financial factors, but also the perceived status of the procedure, the individual's perceived need and finally, the perceived social meaning of DITS and the impact of the expenditure. The following section examines these influences on individuals' decision making and ends by considering how individuals can act as consumers of healthcare.

### Deciding to pay for private healthcare: a matter of price?

For many people, their freedom to choose to purchase private healthcare treatments will be determined by their (in)ability to pay; inequalities in access on the grounds ability to pay are inherent in a private healthcare market. Whereas some participants discussed paying for DITs in instalments in the same way one might finance other expensive household purchases, for others the cost was prohibitive:

I hated it [denture] totally, it was horrible... I was like adamant I was going to get it [DITs] done... [But] it would have cost £6000 and when I got the compensation I got nowhere near the amount that... so I've had to make do with a [removable denture] (F051207;23;Declined)

This woman's tooth loss has a significant negative impact on her daily life, and yet, her preferred treatment option is unavailable to her because it is unaffordable. Inequalities to access and exclusion on the grounds of price may not be problematic for a 'non-essential' item or procedure, but is a concern when it means someone with an apparent disability is unable to access a particular health care treatment because they do not possess the necessary financial resources.

For most people in our sample, deciding to pay for DITs was commonly not simply about affordability, but was often a more complex process, understood by considering the social and healthcare context in which decisions were enacted. Although people in the UK are familiar with contributing towards their NHS primary care dental costs, such costs are relatively modest; paying significant sums of money on healthcare is comparatively uncommon. The following accounts illustrate two contrasting views from our respondents about paying for healthcare:

The original quote I had was about £11,000, when I speak to people they think... you must be crackers doing that... It's funny, people would spend that if they had to do some work on the house or the car, but people are reluctant to spend money on their health. (M050208;50;still deciding)

I think over the years, it's gradually become accepted now, people pay for dental treatment, particularly now because there are not so many NHS dentists. (M110707;51;paid)

Attitudes towards paying for private healthcare treatments are shaped by the social and healthcare context in which those decisions are made [13], and in the UK, the NHS acts to delimit what are considered legitimate healthcare needs [14]. Whilst private procedures are readily available in the UK, the wider context of a universal health care system seems to mean that healthcare not available via the NHS is often viewed, by implication, as an "extra". The fact that DITs are generally available only privately appeared to imbue them, for some people, as being purely about aesthetic need rather than functional necessity. They often seem to be regarded as, akin to private 'cosmetic' dental procedures, rather than an optimal treatment option, let alone a "minimum standard of care" with any associated entitlement.

The NHS is not for a cosmetic sort of thing, it's for looking after your teeth and gums and if you want additional things I think you should pay for it" (M150108;58;paid).

Our data suggest that DITs appear to exist in a contested space, on a continuum from 'functional necessity' to 'cosmetic need'; a continuum which is subject to individual interpretation. The interpretation of the position on this continuum appears to influence individual's decision making. Some respondents who declined treatment alluded to a difference between 'real' functional need and aesthetic want: for them, private DITs seemed a luxury when removable dentures available through 'standard' care could provide a perfectly acceptable solution to tooth loss:

I think dental implants certainly come under the area of what people want, rather than what they need, cos what they need, I guess is a set of dentures. (M140108;34;declined)

It's like teeth, as long as you look reasonably OK and you can open your mouth and smile and they're functional, you can chew your grub [food] and have the occasional steak, if you can do that, great. (M240507;65;declined)

When people did pay for healthcare they appeared usually to need to justify their decision, emphasising their medical and functional needs rather than an aesthetic want.

I couldn't chew properly as well, so it was a necessity really... if I had teeth which would function, I wouldn't have got it done to improve the appearance, so there was a need to get it done, so it wasn't completely cosmetic (M110707;51;paid)

... yeah, you can have it done just like to make you get a nice smile no, no, no I'm having it done because I need to chew (M201107;64;paid)

This echoes the accounts of the UK women in Gimlin's study who, in contrast to her US respondents, carefully constructed their narratives around the 'medical need' - pain or emotional consequence of their current physical state - rather than vanity for their cosmetic procedure [13].

### "Family money"

Rapley [15] has argued that decision making in healthcare is 'distributed': not confined to one interaction or one consultation, and influenced and informed by external factors and actors, as well as by previous knowledge and experience. This notion of distributed decision making seems particularly important in the private healthcare market when decisions could impact on others, specifically family. For example, many of our respondents were retired, and whilst some could afford DITs, the decision to purchase was mediated by the personal and social significance and consequences of the proposed spending on their own future or for that of others. Zelizer [16] argues that domestic money unlike "market" money (a homogenous, neutral means of exchange), is "special" and imbued with personal meaning. For our respondents, their money was often 'finite', it was 'family' money to be shared with a spouse, to help a child to buy a house, or for 'just in case' and depleting it had consequences for others:

If I explain my attitude towards money, I expect I will die before my wife, because of my heart condition... I want to provide enough for her to be able to continue to live more or less at the standard that she is now. (M310707;77;still deciding)

In addition, spending money on DITs could be seen as selfish or frivolous, particularly when people thought about what else the money could be used for; altruism and thrift were important. In the following account, despite wanting DITs, this woman presents DITs as an unnecessary or self-indulgent procedure which would unacceptably reduce collective household finances:

I realised the implications of what I was going to have to take from this household... Because it's a cosmetic thing I think... it's the same as having a facelift you know or a boob job. I feel a little bit selfish I suppose to take that amount of money for myself when it's cosmetic. (F070108;60;declined)

In this instance it is precisely the 'image' or perception of DITs as aesthetic rather than functional which is central to some decision making and determined that the expenditure could not be justified, By contrast, the behaviour of others towards spending could also affect decisions to pay for DITs:

It was our savings for if we ever needed anything, and I felt quite guilty about it but then again, he bought a car for £18,000 so I felt well that was my car. My teeth will hopefully last longer than the car. But [he] was fine about it because he's been through the pain with us [me] and knew that I couldn't eat. (F140607;58;paid)

Even though DITs were this woman's 'car', as we saw earlier in others' accounts, she emphasises her functional need to reinforce that it was not a frivolous expenditure and also cites her husband's support of her spending decision. Family 'approval' seems to be particularly important when deciding to pay for private treatments:

[My daughter] said "Mam, I was talking to my dentist today about you don't like your false teeth and he said, 'Oh, that's old hat, you have to have implants these days,'... and she said 'well my mother's not young' he said, 'oh my father's well in his 80's and he's got implants". So she said, "Mam, that's it, you've got to have implants... The only thing was the money, and my husband said, "I don't care if that's what you want to do, you know, we don't spend money on other things" (F150108;80;paid)

Receiving 'permission' from her daughter and husband enabled this woman to justify her spending on DITs. The importance of receiving lay approval or 'sanctioning' before seeking healthcare is well established [17,18]. Our data suggest that such lay 'permission' or 'support' is also important when one is paying for healthcare.

### Towards consumerism?

Although people in the UK will act as 'consumer' in many marketplaces, private healthcare appears to require a different skill set. Whilst the lay person may find it difficult to choose different healthcare providers in general [18], in private healthcare they have to not only assess the provider but *also *judge the suitability of the product. This may be difficult, because whilst clinicians providing private procedures must act in the patient's best interests, they also have a vested financial interest in promoting their service. To compound this, the established relationship and, most importantly, trust an individual has in their clinician may prevent them seeking out alternative treatments or providers, as [19,20]. Further, evidence suggests that patients are still prevented from acting effectively as consumers in UK private dentistry; partly because there is a lack of information on which to make choices [6]. Our research adds to this discussion and points to only limited consumer engagement in this market, despite respondents paying significant sums for DITs, as the following quotes highlight

He's got "implant" written right across his gate... I thought oh, I hope he knows what's he's doing, you don't go and see other people about it or anything like that you know. I mean you just, you go in cold really and then they do it and that's it, and you just hope to heck it comes out right (M201107;64paid)

The following extracts provide an interesting contrast of consumer behaviour, having had a bad experience with one particular practice this respondent, questioned the qualifications of a subsequent dentist who offered her DITs:

She[receptionist] said 'he has been on a training course', I thought 'well, was it a week, was it a day?

However, later she explains how she came to choose her ultimate provider:

I was out with the dogs and I was talking to this chap. I told him what had happened and he said oh you want to try this chap in *[town*], he's pretty good." (F221107;68;paid).

Whilst some 'hoped' everything would work out, we did find evidence to suggest that private dentistry, or at least the market of DITs, may be developing so that people are being treated more like consumers. Almost all respondents who considered paying for DITs were given an individualised written treatment plan and described receiving very clear information. However, respondents rarely reported receiving information about other kinds of DITs that the dentist did not provide, but others might.

He never mentioned[other options], these were the ones he told me about these were the ones he gave me all the information on, there was no one, two, three, there was no alternative (F210607;60;paid)

Our data also suggest that some people are beginning to adopt some of aspects of consumerism seen in other markets. For example, when people purchase expensive goods they expect a level of reliability and guarantee and transferred this expectation to DITs. A perceived unacceptable guarantee could lead some, like this woman, to decline treatment:

I wasn't prepared to put myself through it, without a total guarantee. I know, any kind of operation, I know there is never a total guarantee but, you know what I mean, I would want a higher percentage rate if you like. (F220108;52;declined)

Similarly, in the next quote, this man describes challenging the quote he was given, citing his own internet based research to evaluate appropriateness of price he was being quoted by his dentist.

I could prove to him with all my research that I could get implants or have my teeth done for half or even a third of the price that he was offering... he recommended six which was better... and a bit more realistic. (M120707;65;still deciding)

This was not an isolated case; internet based research was quite common and some people did 'shop around' on-line. However, internet based information can be confusing, is far from standardised and often driven by the self-interest of providers. Furthermore there is rarely any objective indication of the skill, experience or ability of the clinician. It is difficult to know how the patient charged with acting as a healthcare consumer is supposed to make sense of all the information available or make effective comparisons between providers, and more research is needed to examine this issue further.

## Conclusions

We know relatively little about how decisions about treatments and costs are negotiated within a private healthcare context, particularly where there is no third party to oversee costs and quality. This paper has shed some light on this hitherto under researched area and highlighted some areas for further exploration. However, it is important to acknowledge that the data on which this discussion are based are drawn from a qualitative study located in one particular area of the UK, as such they are based on a relatively small number of people's experiences. That said, the aim of qualitative research is never to develop statistically generalisable findings but rather theoretical insights. Whilst this research was conducted in the UK NHS, there are a myriad of other developed oral healthcare systems that use private insurance systems, or some form of social insurance. Like the UK, these often demand out of pocket payments or co-payments for some expensive interventions such as implants. Where substantial direct payments are made, irrespective of the background insurance mechanism, similar decision-making processes might be expected to take place, presumably modified by local factors such as cost, income and culture.

Accepting the limitations outlined above, however, we believe that through examining the accounts of people who considered pursuing DITs in primary care dentistry, this paper provides valuable insights into how people understand, rationalise and engage with the choice of payment for expensive healthcare procedures. For some it is simply about affordability; whereas services provided under the National Health Service can be heavily or completely subsidised to mitigate the risk of inequalities in oral health care related to low income. Services that are only provided privately depend to a large degree on the ability to pay, and inequalities in access to such private health care are therefore inherent, and this is compounded when such access was being sought for functional reasons.

However, our data suggest that, deciding to pay for treatment is not simply about ability to pay, but is mediated by other factors. Our respondents' accounts illustrate that individuals must have a perceived 'need' which they can justify - either functional or aesthetic, but more commonly a combination of both with the emphasis on functional in this context. They must regard the treatment as having improved utility compared to other options and its benefits to the individual must be apparent; like any consumer, individuals make their own judgements about the 'worth' of a treatment in terms of what they are likely to gain, relative to its cost. Our research also suggests that deciding to pay for healthcare is a distributed decision and not merely an individual one; spending is imbued with personal, social and moral significance and must be seen, and presented, as a legitimate use of finite resources. Underpinning the decision, it appears that the status of the procedure is important both to the individual and to others. There has been a growth in the UK private cosmetic healthcare market in recent years and, despite significant functional disability, some people appear to see DITs as a cosmetic luxury because they are only available privately.

The data from this study raises some concerns about how well patients are able to act as informed purchasers and consumers of healthcare. If more healthcare becomes available purely as a personal transaction, our data suggest that market forces alone may not be enough to manage supply and demand. For the market to work efficiently, consumers, who are also patients, need to be enabled to make informed decisions based on accurate information, perhaps through the 'visible hand' of the state empowering them as consumers [2]. Our respondents' accounts appear to show that consumer knowledge of treatment options and providers was far from optimum and poorly supported. Within a private healthcare market the individual clinician-patient relationship is paramount. Our data suggest that personal recommendation, existing relationship and trust with an individual clinician was a significant factor in deciding whether or not to pursue DITs and the choice of provider. If this is indeed the case, attention needs to be given to how to protect patients from exploitation in a high cost market, and specifically how they can be protected from receiving inappropriate, substandard or overpriced care. As private healthcare markets continue to expand and chronic disease and disability become the dominant features in our ageing populations, it is likely that more people will pursue private healthcare procedures at the fringes of clinical necessity. Our study suggests that individuals need to become adept at balancing the dual and sometimes contradictory roles of, patient and consumer if the private healthcare market is to function efficiently. This paper has further offered some initial suggestions as to the complex factors influencing patient decision making in a private health care market. Further research could usefully investigate whether these factors apply in other conditions and contexts.

## Abbreviations

NHS: UK National Health Service; DITs: Dental implant treatments for toothloss. Traditionally removable full or partial dentures have been used. DITs involve the placement of titanium implants into the jaw on to which either removable or fixed dentures are placed; ISODs: Implant supported over dentures. A specific kind of DITs involving the placement of removable Dentures attached to implants fixed in the jaw.

## Competing interests

The authors declare that they have no competing interests.

## Authors' contributions

CE in collaboration with CD and JS and the wider research team designed the original study and obtained funding. CE and NR developed the interview guides, NR conducted the interviews and analysis was conducted jointly by both CE and NR. Final interpretation was undertaken jointly by all authors. CE wrote the first draft of this paper, NR, CD and JS commented on subsequent drafts. All authors approved the final version. All authors are guarantors and accept full responsibility for the conduct of the study and the contents of the paper.

## Pre-publication history

The pre-publication history for this paper can be accessed here:

http://www.biomedcentral.com/1472-6963/12/53/prepub
